# Interplay Between Composition and Cycling Performance of Pre-Lithiated SiO_x_-Si-C Composite Anodes for Lithium–Sulfur Full Cells

**DOI:** 10.3390/ma18051053

**Published:** 2025-02-27

**Authors:** Swamickan Sathya, Ramasamy Santhosh Kumar, Sara Garcia-Ballesteros, Federico Bella, Dong Jin Yoo, Arul Manuel Stephan

**Affiliations:** 1 Electrochemical Power Sources Division, CSIR-Central Electrochemical Research Institute, Karaikudi 630003, India; smsathya09@gmail.com; 2 Department of Life Science, Department of Energy Storage/Conversion Engineering of Graduate School (BK21 FOUR), Hydrogen and Fuel Cell Research Center, Jeonbuk National University, 567 Baekje-daero, Jeonju 54896, Jeollabuk-do, Republic of Korea; vedhasanthosh9944@gmail.com; 3 Department of Applied Science and Technology, Politecnico di Torino, Corso Duca Degli Abruzzi 24, 10129 Torino, Italy; sara.garcia@polito.it

**Keywords:** silicon sub-oxides, discharge capacity, pre-lithiation, lithium–sulfur batteries, solid electrolyte interphase

## Abstract

Although silicon-based anodes have been identified as a potential alternative to conventional graphite anodes, the huge volume change (approximately 300%) that occurs in silicon while cycling still impedes this system from practical applications. In the case of silicon-suboxide (SiO_x_)-based anode materials, both Li_2_O and LiSiO_4_ are formed during the initial lithiation processes and act as a natural volume buffer matrix to accommodate volume changes and the formation of a stable SEI layer, which improves the cyclability and capacity retention. In this study, a series of SiO_x_/Si/C-based electrodes composed of different amorphous SiO_x_, Si, and graphitic carbon contents were prepared. Among the various investigated compositions, the electrode with a ratio of SiO_x_-Si-C equal to 70:12.5:12.5 was found to be optimal in terms of discharge capacity. This promising electrode was pre-lithiated prior to cycling. Finally, 2032-type lithium–sulfur (Li-S) coin cells composed of a S-C/SiO_x_-Si-C (pre-lithiated) configuration were assembled and their cycling performances are reported.

## 1. Introduction

The implementation of electric vehicles, depletion of fossil fuels, and the demand for large-scale electrochemical energy storage devices have triggered the requirement for long cycle lives and high-energy-density rechargeable batteries with better safety and reliability [1,2]. Owing to their unique characteristics, such as their low cost, high abundance of elemental sulfur, lower toxicity, and high theoretical capacity (1672 mAh g^−1^) and specific energy (2600 Wh kg^−1^), lithium–sulfur (Li-S) batteries have been identified as a possible next-generation energy storage technology [3,4]. However, the undesirable issues such as self-discharge, the transport of lithium polysulfides between the electrodes, and the intrinsic insulating nature of elemental sulfur (5 × 10^−30^ S cm^−1^ at 25 °C) hamper Li-S batteries from immediate commercialization [5,6,7]. The engineering of novel electrode materials, such as composite sulfur cathodes, modification of liquid electrolytes with different additives and lithium salt, and protection of the lithium anode (artificial solid electrolyte interphase—SEI) can appreciably enhance the electrochemical performances of Li-S batteries [8]. The unhindered formation of lithium dendrites as a result of irregular deposition and stripping processes while cycling is another major issue of Li-S batteries, which results in poor Columbic efficiency [7]. More importantly, when the composite sulfur cathode is coupled with a lithium metal anode, the deposition of lithium polysulfides on lithium corrodes its surface and eventually reduces the cycle life of Li-S batteries [9,10].

To address these challenges, a variety of metal alloy anodes has been examined. In this regard, group IVA elements such as silicon, germanium, and tin are considered as potential candidates for next-generation batteries [11,12]. Among them, silicon has been considered the most attractive anode material due to its appealing features, such as its high theoretical capacity (4200 mAh g^−1^ for Li_4.4_Si), abundance, low cost, and favorable working voltage [13]. However, silicon anodes possess undesirable issues such as huge volume changes (300%) during lithium-ion insertion/extraction processes, low electrical conductivity, and an unstable SEI, resulting in mechanical stresses and fracture of the electrode as well poor cycling performances [14]. Volume changes cause silicon particles to pulverize, which consumes a significant amount of electrolyte in the process of creating a new SEI layer, and increased cell impedance, which further reduces battery performance [15]. Various strategies have been explored to overcome these challenges, including (i) the adoption of different nanostructures [15,16], such as silicon nanowires, nanotubes, and nanoparticles; (ii) mesopore designs; and (iii) the use of electrolyte additives [17] and (iv) bi-functional binders [18]. Recently, silicon suboxides (SiO_x_, 0 < x < 2) were proposed as potential anode materials due to their abundant reserves, ability to operate at favorably lower potentials, low cost, facile synthesis, and environmental friendliness. In addition, these materials offer a theoretical capacity of 2615 mAh g^−1^ and improved cycling capability compared to silicon, with smaller volume changes (~160%) upon electrochemical cycling [19]. The non-stoichiometric SiO_x_ can be mass-produced through a vapor deposition method by combining SiO_2_ with gaseous SiO formed by heating SiO_2_ at high temperatures [20]. The formation of Li_2_O and LiSiO_4_ during the initial lithiation process is due to the reaction of lithium ions with SiO_x_, which decreases the specific capacity. Nevertheless, the formed Li_2_O and LiSiO_4_ act as natural volume buffer matrices and accommodate volume variations, which promotes the cyclability and capacity retention [21]. Even though the formation of Li_2_O and LiSiO_4_ is advantageous to prevent pulverization of SiO_x_ complexes, several crucial issues, such as an inadequate rate capability, low Coulombic efficiency, and an extremely short cycling life at elevated current rates, occur due to the low electrical conductivity (6.7 × 10^−4^ S cm^−1^) and poor lithium-ion diffusion and must be resolved [22,23]. To alleviate these issues, combining SiO_x_ with conductive layers of carbon or a carbon matrix provides high conductivity and can significantly improve the charge transfer capability.

Tang et al. studied the electrochemical properties of amorphous SiO_x_ by varying the oxygen content [24]. The authors found that SiO_x_ with a low oxygen concentration had improved reversibility and initial Coulombic efficiency (ICE), but decreased capacity retention after cycling. Yan et al. created a SiO_x_/SiO_y_ rolled-up bi-layer nano-membrane anode in order to combine the benefits of the two parts. The central silicon-rich SiO_y_ layer (y = 0.5) has a large volume for lithium-ion storage provided by the electrochemically active silicon domains, and the outer oxygen-rich SiO_x_ layer (x = 1.85) acts as a strain buffer/mechanical support layer for volume change accommodation [25]. Sun et al. used a straightforward argon/hydrogen reduction process to create a low-cost, high-capacity SiO_x_/C@graphite (SCG) composites from oat husks. The authors also investigated the impact of graphite on the electrochemical properties of SCG composites. The SCG-1 composite (where 1 signifies a 1:1 ratio) showed a particular ability of 809.5 mAh g^−1^ at 0.5 A g^−1^, even after the 250th cycle, along with a high-rate ability of 479.7 mAh g^−1^ at 1 A g^−1^ after the 200th cycle in lithium-ion batteries [26]. Hagen and co-workers [27] prevented the formation of lithium dendrites in Li-S batteries using pre-lithiated silicon nanowires. The achieved an energy density of about 300 Wh kg^−1^ with a silicon anode and a sulfur cathode, which was found to be higher than that of commercialized systems.

In the present work, in order to identify a safe and reliable lithium metal-free anode for Li-S full cells, a series of electrodes were prepared with different amounts of amorphous SiO_x_, silicon (Si), and graphitic carbon (as a conductive additive). Silicon nanoparticles are expected to accommodate the huge volume change that occurs during lithium alloying and de-alloying processes. Among the various investigated compositions, the electrode composed of a ratio of SiO_x_-Si-C at 70:12.5:12.5 was found to be optimal in terms of discharge capacity with a lithium half-cell configuration. This promising electrode was finally pre-lithiated using the direct contact method prior to cycling. Finally, CR2032-type coin cells composed of a S-C/SiO_x_-Si-C (pre-lithiated) configuration were assembled and their cycling performances are reported.

## 2. Experimental

### 2.1. Materials

Silicon suboxide (SiO_x_ with a particle size of 5 μm; Japan’s Osaka Titanium Corporation), silicon (particle size of 10 nm; Alfa Aesar, Leicestershire, UK), lithium bis(trifluoromethanesulfonyl)imide (LiTFSI), and polyacrylic acid (PAA, Mw ∼4,000,000; Sigma-Aldrich, St. Louis, MO, USA) were used as obtained without further purification. A liquid electrolyte, that was not watery, containing LiTFSI 1 M in battery-grade diluters like 1,3-dioxolane (Spectrochem, Mumbai, India) and tetraethylene glycol dimethyl ether (Spectrochem), at a ratio of 1:1 (*v/v*), and fluoroethylene carbonate (Sigma-Aldrich) 5 wt% was formulated. Graphitic carbon (Alfa Aesar, UK) was used as a conductive carbon source. The sulfur–carbon (S-C) composite cathode was prepared using elemental sulfur (Sigma-Aldrich, USA) and multi-walled carbon nanotubes (MWCNTs; Sigma-Aldrich, USA). For the S-C/SiO_x_-Si-C full-cell studies, the non-aqueous liquid electrolyte was enriched with 1% LiNO_3_ as an additive.

The SiO_x_ material used in this work was purchased, but it can be synthesized using two main methods: (i) the sol–gel process that involves the hydrolysis and condensation of silicon alkoxides in the presence of water and a catalyst, leading to the formation of a colloidal suspension (sol) that transitions into a gel; this approach allows for precise control over the particle size and morphology of the SiO_x_ materials [28] and (ii) precipitation methods, where SiO_x_ nanoparticles are produced by inducing the precipitation of silica from a solution containing silicon precursors. Factors such as temperature, pH, and reactant concentrations play crucial roles in determining the characteristics of the resulting SiO_x_ particles [29]. A further process is named the Stöber method, which is a specific type of sol–gel method that enables the synthesis of monodisperse, spherical SiO_x_ particles through the controlled hydrolysis of TEOS in an alcoholic medium with ammonia as a catalyst; this technique is well known for producing uniform SiO_x_ particles with tunable sizes [30]. 

The combined effects of morphology and composition play a crucial role in the lithiation performance of SiO_x_-based anodes. Morphology influences lithium-ion diffusion, mechanical stability, and electrode–electrolyte interactions, while the composition determines the electrochemical characteristics, such as capacity, conductivity, and volume expansion management.

### 2.2. Preparation and Electrochemical Characterization of the Electrodes

The slurry for the working electrode was prepared with different amounts of SiO_x_, silicon, and graphitic carbon, as shown in Table 1. Lithiated PAA (Li-PAA) was used as a binder, using deionized water to make an emulsion. The electrodes were made by applying the slurry onto copper foil and drying it for 12 h at 100 °C. The S-C composite cathode was prepared with elemental sulfur and MWCNTs at a mass ratio of 70:30, and then it was ground for 1 h. The composite was maintained at 155 °C for 12 h under a N_2_ atmosphere. Regarding the choice of the binder, the reader can refer to our former manuscript, where we investigated SiO_x_-Si-C electrodes based on different binders (i.e., PAA, carboxyl methyl cellulose, and their blends) [31]. 

For electrochemical measurements, these electrodes were assembled into CR2032-type coin cells using a Celgard 2320 separator (Bangalore, India) and lithium foil Sigma Aldrich) as the counter electrode. The liquid electrolyte detailed in the experimental section was adjusted to form a stable SEI layer [32]. The electrochemical cells were assembled in a glove box filled with high-purity argon (99.9995%). Using a VSP3 Biologic (Seyssinet-Pariset, France) electrochemical workstation, measurements of electrochemical impedance spectroscopy (EIS) were performed in the frequency range of 1 MHz to 100 MHz. The battery cycle life tester was used for galvanostatic charge–discharge studies in the potential range of 0.005–2.5 V at a rate of 0.1C.

Between the pure and cycled electrodes, the change in electrode morphology was examined (100 cycles) using scanning electron microscopy (SEM; TESCAN VEGA3; USA) with an accelerating voltage of 15 kV. X-ray photoelectron spectroscopy (XPS; Thermo Scientific, Waltham, MA, USA) analysis of the cycled samples was carried out using an Al-Kα X-ray radiation source (photon energy 1486.7 eV) with an emission angle of 45°. CASA was used for additional analyses after Thermo Advantage V5.9925 software was used to collect the data. The C–C peak at 284.4 eV was used to calibrate the data to calculate the binding energies of each of the current elements. Peak shapes of 10% Lorentzian and 90% Gaussian Voigt were used to fit the curves. To neutralize the charge, an electron flood gun was used. The change in morphology of the electrode was studied on pristine and pre-lithiated samples using field emission scanning electron microscopy (FESEM; SUPRA 55VP; Berlin, Germany) with an accelerating voltage of 5 kV. High-resolution transmission electron microscopy (HR-TEM; Thermo Scientific FEI Tecnai G2 F20) was employed to examine the structural alterations that occurred inside the SiO_x_-Si anodes during lithiation.

## 3. Results and Discussion

### 3.1. Charge–Discharge Studies

**Figure 1:**
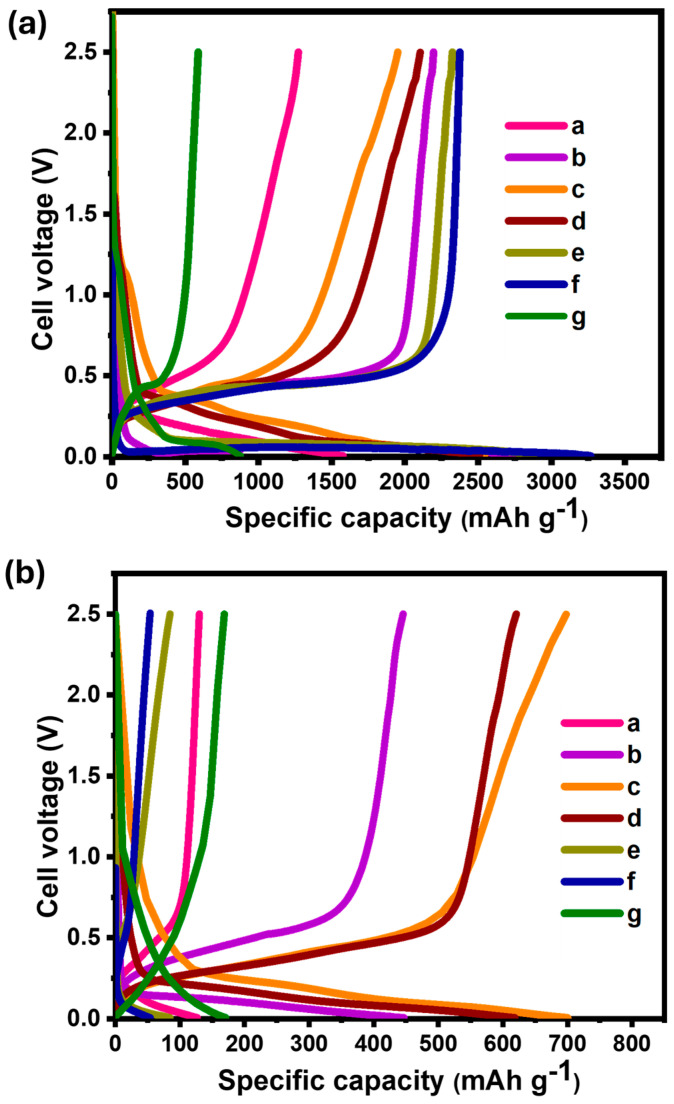
Charge–discharge profiles of Li/SiO_x_-Si-C cells with different compositions (a–g), the details of which are shown in Table 1. Panel (**a**) shows the data for the 1st cycle and panel (**b**) shows the data for the 100th cycle.

Figure S1a–g displays the galvanostatic charge–discharge curves of Li/SiO_x_-Si-C lithium cells with varying SiO_x_, silicon, and carbon contents at a rate of 0.1C. The voltage profiles that correspond to the initial discharge (lithiation) of SiO_x_-Si-C were consistent regardless of the electrode composition. This suggests that, through the alloying and de-alloying processes, the composition had no discernible impact on the formation of the SEI layer. Li/SiO_x_-Si-C cells with 85, 75, 70, 65, and 10% SiO_x_ had an irreversible capacity loss of 40, 30, 19, 32, and 18%, respectively. The discharge capacity vs. number of cycle Li/SiO_x_-Si-C cells for the different compositions and their corresponding Coulombic efficiency are shown in Figure S4a–g, respectively. The Li/SiO_x_-Si-C electrode in a lithium cell with 70% SiO_x_ offered the highest discharge capacity among the cells with different contents of silicon and graphitic carbon. The Li/SiO_x_-Si-C cell containing 85% SiO_x_ provided a capacity for an initial discharge of 1272 and 129 mAh g^−1^ on the 100th cycle, with almost 99% Coulombic efficiency (Figure S4a). The discharge capacity exponentially decreased when the content of SiO_x_ was reduced to 10%. Figure 1 shows the data recorded at the 1st and 100th cycles.

A Li/SiO_x_-Si-C cell with 75% silicon (sample e) offered the highest discharge capacity of 2323 mAh g^−1^ on its first cycle, and a drastic reduction was observed after 100 cycles, when it reached 85 mAh g^−1^. This observation was attributed to the huge volume change that typically occurs in silicon-based anode materials. A similar result was observed when the content of silicon was further increased to 85% (sample f, Figure 1). The Li/SiO_x_-Si-C cell with 10% silicon and 75% graphitic carbon (sample b) showed a discharge capacity of 588 mAh g^−1^. The observed higher values were due to the added SiO_x_ and Si particles. At the end of 100th cycle, the Li/SiO_x_-Si-C cell was able to deliver 169 mAh g^−1^ with 99% Coulombic efficiency. Accordingly, variations in the chemical compositions (both organic and inorganic) of the SEI layer, which differed with the material formulations, may be the cause for the variations in the cell cycling performance. In order to provide more insight into the geometrical and structural characteristics, the cycled electrodes were disassembled into a glove box filled with argon and analyzed using FESEM and XPS.

### 3.2. Morphological Characterization

**Figure 2:**
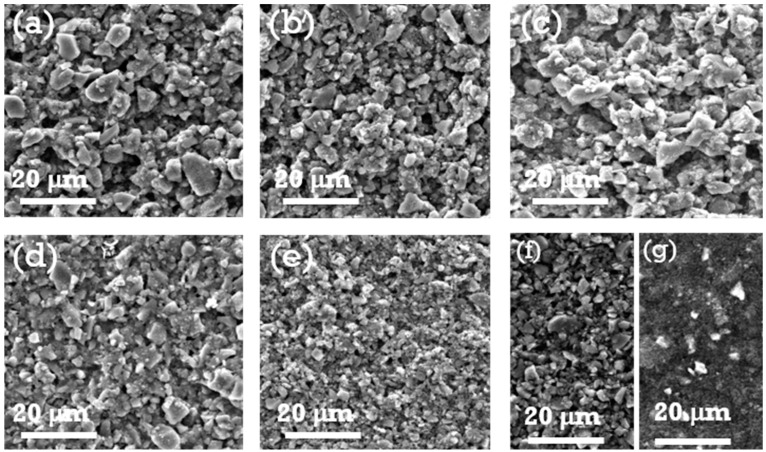
FESEM images of SiO_x_-Si-C electrodes with different compositions (**a**–**g**), the details of which are shown in Table 1, before being cycled.

**Figure 3:**
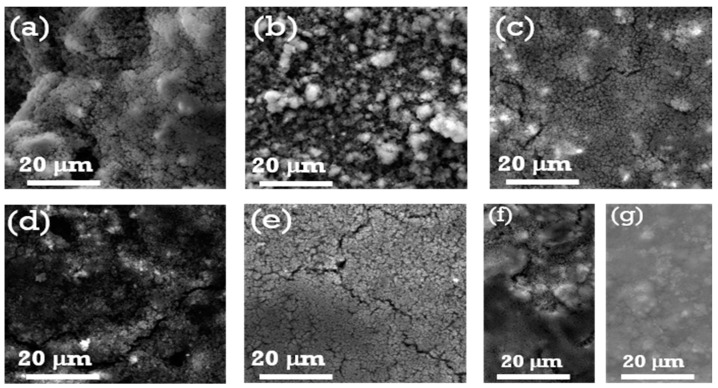
FESEM images of SiO_x_-Si-C electrodes with different compositions (**a**–**g**), the details of which are shown in Table 1, after being cycled 100 times.

Figure 2 and Figure 3 show FESEM images that reveal the surface morphologies of the SiO_x_-Si-C electrodes with different compositions. The surface morphologies of the electrodes varied based on the composition of the electrodes. The as-prepared composite electrodes with a high amount of SiO_x_ dispersed with acetylene black and silicon particles exhibited a rough surface with large voids (Figure 2a–d). On the other hand, a smooth surface morphology with fewer voids were observed for the samples with higher contents of Si and C particles (Figure 2e,f). A similar surface morphology was observed for the sample with 75% graphite (Figure 2g). Upon cycling, the voids in the electrodes disappeared, and the surfaces became dense and remained intact; however, cracks were detected on their surfaces. For the samples with larger amounts of graphite (Figure 3g), no significant changes were observed in surface morphology, indicating that the graphite samples did not undergo any noticeable volume change in the first 100 cycles. It is worth mentioning that the samples with 70, 75, and 85% SiO_x_ showed cracks on their surfaces and the formation of discontinuous islands. A huge reduction in the discharge capacity values upon cycling was observed and can be attributed to the volume changes that occurred on the electrode surface. In addition to this, the reduction in the values of the discharge capacity can be presumably related to more irreversible phase transitions that occurred, namely the formation of lithium silicate and its subsequent volume expansion and accumulation of SiO_x_/C particles [33].

### 3.3. Cyclic Voltammetry Studies

The successive cyclic voltammograms of Li/SiO_x_-Si-C half-cells with electrodes with the different compositions are shown in Figure S3a–g. These cyclic voltammograms of the Li/SiO_x_-Si-C cells make it clear that the processes of alloying and de-alloying lithium–silicon (Li-Si) are entirely reversible. The Li-Si alloying reactions were attributed to the current peaks that were seen at 0.16 and 0.03 V on the initial cathodic sweep [34]. The Li-Si de-alloying process corresponded to the anodic current peaks, which emerged at 0.46 and 0.30 V, respectively. The peak at 0.35 V for the 85% Si Li/SiO_x_-Si-C cell vanished in the ensuing cycles, signifying an irreversible process that transformed elemental silicon into lithium silicates (Li_2_Si_2_O_5_, Li_2_SiO_3_, and Li_4_SiO_4_) [34]. In contrast, the peaks were unaltered for the Li/SiO_x_ -Si-C cell possessing 85% SiO_x_. For the Li/SiO_x_ -Si-C cell with 75% graphitic carbon, anodic peaks appeared at 0.19, 0.34, 1.2, and 1.48 V (Figure S3g), representing the typical characteristics of carbon. The magnitude of the voltammetry peaks increased and decreased when the SiO_x_ content was high, while a dissimilar trend was observed for higher silicon contents. The pulverizing and amorphization of silicon upon cycling was identified as the cause for the rise in the number of oxidation and reduction peaks as a function of cycles for the electrodes with a higher content of silicon [35].

### 3.4. Impedance Analysis

It is commonly known that EIS is a potential tool for examining the many intricated mechanisms of both Li-S and Li-ion batteries while cycling [36,37]. EIS is commonly used for probing Li-S battery electrolyte compositions [38,39] and their performances [40]. The semicircles of the Nyquist plots are well developed at lower potentials. The high-to-medium frequency range relates to charge transfer resistance; conversely, low-frequency lines become extremely steep and describe the features of the electrode/electrolyte interface. The morphologies of silicon-based anode components are generally more complex, and it is time-consuming to determine the semicircles with precision [41].

**Figure 4:**
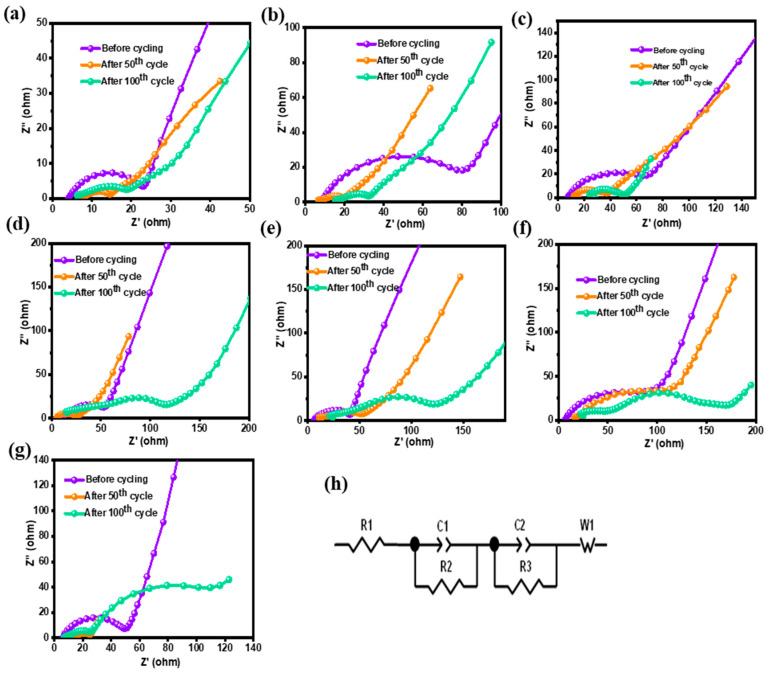
Nyquist plots of Li/SiO_x_-Si-C cells with different compositions (**a**–**g**), the details of which are shown in Table 1, after different numbers of cycles. (**h**) Equivalent circuit used for data fitting.

The low frequency signal portrays the diffusion processes, such as the solid-state diffusion of lithium ions into silicon, whereas the high frequency signal depicts the surface chemistry of the silicon-based anode. The impedance of these electrodes displayed a capacitive characteristic at very low frequencies because of the Li-Si alloy’s potential dependency on the lithium concentration. The Nyquist plots of the Li/SiO_x_-Si-C half-cells with different amounts of SiO_x_, silicon, and graphitic carbon before cycling and after 50 and 100 cycles are shown in Figure 4a–g. The Li/SiO_x_-Si-C cell with 85% SiO_x_ offered the lowest R_s_ values of 4, 6, and 5 Ω before, and after 50 and 100 cycles with corresponding R_ct_ values of 20, 5, and 8 Ω, respectively. As a result of cycling, the gradient reduced, suggesting that lithium ions were diffusing throughout the active substance [42]. Additionally, the resistance of the recently created SEI layer (RSEI) is represented by the semicircle diameter in the medium-frequency region [42]. Of all the systems that were studied, the total resistance (R_s_ + R_ct_ + R_SEI_) of the Li/SiO_x_-Si-C cell with 85% SiO_x_ was the lowest, i.e., 18 Ω after 100 cycles. In contrast, the Li/SiO_x_-Si-C cell with 85% silicon and 75% graphite showed values of 137 and 21 Ω, respectively, after 100 cycles. The higher R_SEI_ values after 100 cycles were attributed to the massive increase in the silicon-based electrode volume, which resulted in cracking of the electrode surface and poor cycling performance of the silicon-based anodes (see Table S2). These results are in agreement with the FESEM images (Figure 2 and Figure 3) and with the charge–discharge study results (Figure 1).

### 3.5. XPS Studies

**Figure 5:**
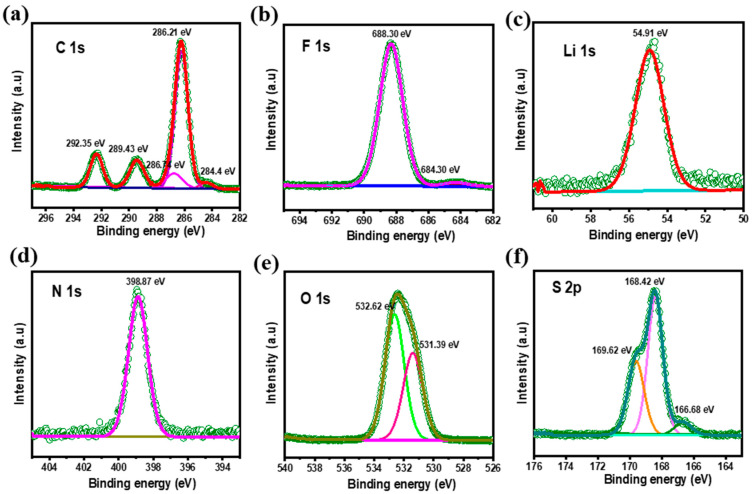
XPS spectra of sample ‘e’ (75% Si + 10% SiO_x_ + 10% C) after 100 cycles: C 1s (**a**), F 1s (**b**), Li 1s (**c**), N 1s (**d**), O 1s (**e**), S 2p (**f**).

The electrochemical performance of Li-S batteries is largely dependent on the surface properties of both the anode and cathode. In order to analyze the surface properties of cycled SiO_x_-Si-C electrodes, the cells were carefully disassembled in an argon-filled glove box and were subsequently washed with solvents and vacuum-dried to eliminate any residual LiTFSI. In the present work, the SiO_x_-Si-C composite electrodes with 75% Si and 75% SiO_x_ were analyzed and the results are depicted in Figure 5 and Figure S4, respectively.

In our earlier report [32], we analyzed the surface films formed on SiO_x_ (70%)-based electrodes; however, no discernible changes could be seen when the SiO_x_ content was increased to 75% (Figure S5). The XPS spectra relative to the C 1s, O 1s, and Li 1s signals for an SiO_x_-Si-C electrode with 75% Si are depicted in Figure 5. The C 1s signals observed at 284.4, 286.21, and 289.43 eV remained attributed to C–C, C–O, and C=O bonds, respectively. Higher C–O and C=O concentrations were seen in the deconvoluted C 1 s spectra for the electrolytes containing carbonate. The production of C-F is represented by the peak detected at 292.35 eV (i.e., –CF_2_) [43,44,45,46,47,48].

Regardless the compositions, the emergence of F 1 s peaks at 684.3 eV and 688.3 eV were seen, which indicate the formation of organic fluorides (C-F bonds) and metal fluorides (LiF) on the electrode surface, respectively. For the electrode with 75% SiO_x_, an additional peak at 689.09 eV was seen (Figure S5), signifying the formation of –CF_2_. In Figure 5, the Li 1 s spectrum shows a broad peak at 54.9 eV, which was attributed to the formation of lithium hydroxide or alkoxide. The peak observed at 398.87 eV represents the formation of Si_3_N_4_. The O1s spectrum of the SiO_x_-Si-C composite electrode cycled with 75% Si and SiO_x_ shows deconvoluted peaks at 531.3 and 532.62 eV, which were assigned to metal carbonate and carbonyl, or organic C=O bonds [49]. The peaks observed at 166.68 and 168.42 eV indicate lithium sulfate (Li_2_SO_3_) and metal sulfate, respectively.

### 3.6. Pre-Lithiation Studies

In general, pre-lithiated electrodes have a superior rate capability compared to un-pre-lithiated ones, and this is attributed to the reduction in the impedance values [50]. In particular, pre-lithiation (PRL) of silicon-based anodes can result in pre-volume expansion, which lessens the proportional change of the silicon during cycling. This will further reduce the cracking or pulverizing of silicon particles and may improve the electrode mechanical stability [51,52]. Winter et al. [53] extensively reviewed the different techniques that are widely employed for the PRL of silicon anode materials, including electrochemical techniques, direct contact with lithium metal, or PRL using electrode additives like stabilized lithium metal powders and superionic conductors.

In the present work, we used the direct contact method, as it is easy to perform and inexpensive. For comparison, the SiO_x_-Si-C electrode was also pre-lithiated using an electrochemical method and its cycling performance was evaluated. As previously noted [53], a longer lithiation time, besides improving the quantity of lithium in the silicon powdered form, results in poor cyclability [52,53,54]. However, despite the fact that the lithiation of the silicon anode for 30 min had less lithiation than the sample treated for 60 min, using this contact duration still achieved the theoretical sulfur cathode capacity of a Li-S full cell. As a result, in the present work, the SiO_x_-Si-C electrode was lithiated for 30 min for the full-cell investigations.

**Figure 6:**
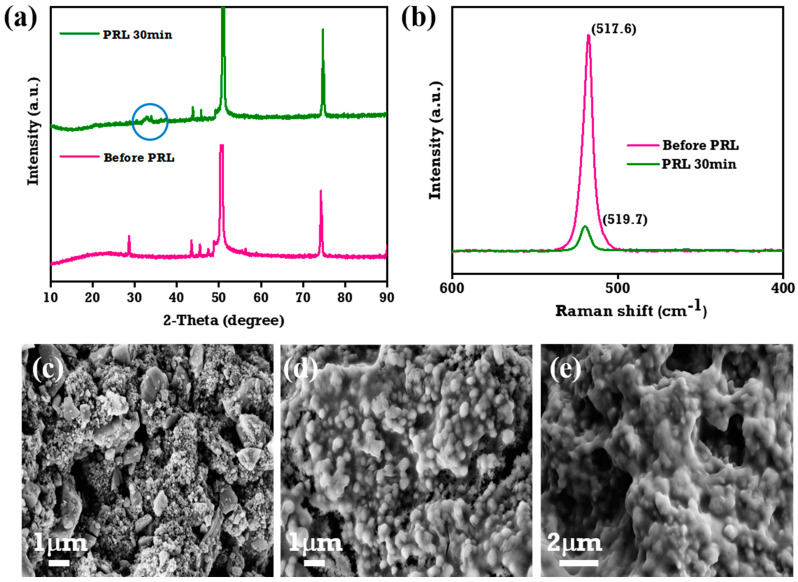
(**a**) XRD and (**b**) Raman spectra of SiO_x_-Si-C electrodes before and after lithiation. Surface morphology of SiO_x_-Si-C electrodes (**c**) before lithiation, (**d**) 30 min after lithiation, and (**e**) 45 min after lithiation.

The XRD patterns of lithiated SiO_x_-Si-C anodes in a LiTFSI 1 M solution of electrolyte in close contact (direct contact method) with lithium foil without a separator are displayed in Figure 6a [50,55]. The diffraction peaks at 27°, 43.7°, and 51° represent the Cu current of the anode, while the peaks at 48° and 56° corresponded to silicon. However, the peak at 27° was insignificant due to the overlapping of the copper and Si (111) planes. The appearance of a peak at 2θ = 33.5° indicates that the lithiated composite anode retained its crystal structure even after lithiation, and it also exhibited the formation of a Li_x_Si alloy (circled in Figure 6a) [52].

The Raman spectrum of the PRL SiO_x_-Si-C composite anode is depicted in Figure 6b. The intensity of the peak at 517.6 cm^−1^, which is a characteristic of crystalline silicon, was reduced upon lithiation. In agreement with the XRD data, the SiO_x_-Si-C composite anode that was lithiated for 30 min exhibited a lower peak intensity than un-pre-lithiated anode, which suggests a change of the crystalline phase into an amorphous phase [52]. Figure 6c–e shows the FESEM images of the SiO_x_-Si-C composite anodes before and after PRL for 30 and 45 min, respectively. As seen in Figure 6d,e, the images demonstrate that the lithiated SiO_x_-Si-C composition anodes’ surface shape was preserved even after lithiation. The insertion of lithium ions caused volume expansion, which resulted in a small increase in the mean diameter of the lithiated silicon particles from 100 nm to 400 nm following the PRL process [50,56].

**Figure 7:**
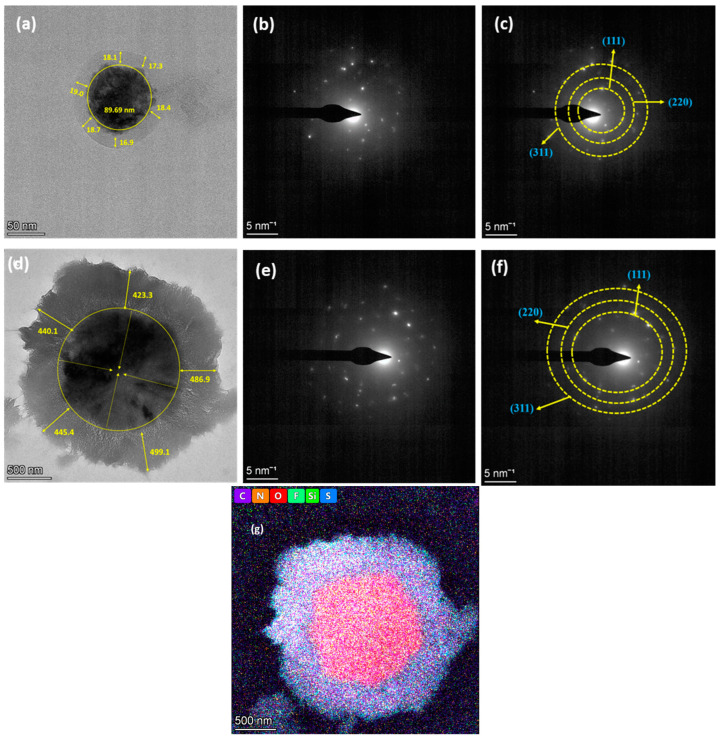
HR-TEM micrographs of samples (**a**–**c**) before and (**d**–**f**) after PRL; (**g**) elemental mapping after lithiation for 30 min using a direct contact method.

Further investigation of the internal structural alterations in the SiO_x_-Si particles during lithiation was conducted using HR-TEM. The pristine silicon nanopowders covered with carbon were completely crystalline and had smooth surfaces (Figure 7a). The size of the carbon-coated silicon particles was calculated to be 90 nm, despite the fact that their thickness was more or less uniform. The selected area electron diffraction (SAED) patterns in the HR-TEM images were in agreement with the XRD data for the Si (111), (220), and (311) planes (Figure 7b,c). These results are in accordance with earlier reports [35,57,58,59]. Upon lithiation, the surface of the coated area slightly changed with the formation of the SEI layer (Figure 7d). The electrode after PRL also showed the presence of the Si (111), (220), and (311) planes (Figure 7e,f). The existence of carbon, nitrogen, oxygen, fluorine, silicon, and sulfur in the electrode after PRL was identified from elemental mapping (Figure 7g).

**Figure 8:**
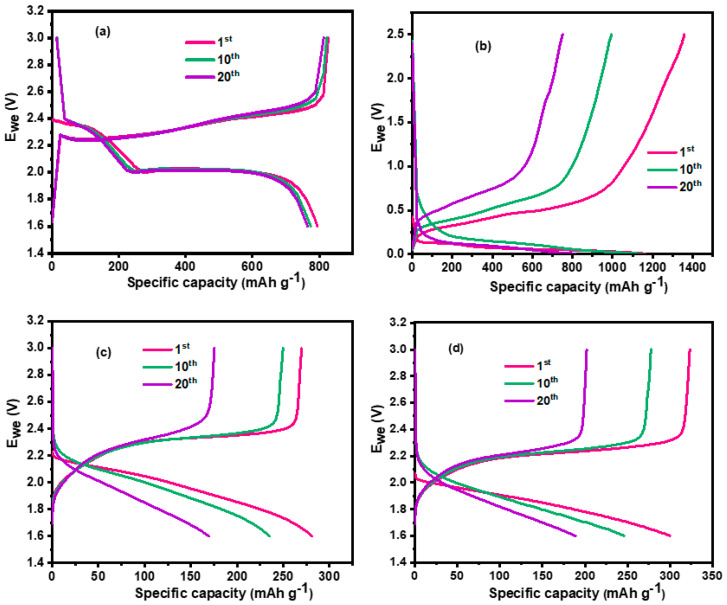
Charge–discharge profiles of (**a**) Li-S half-cell, (**b**) Li-SiO_x_-Si-C half-cell, (**c**) S-C/SiO_x_-Si-C full cell (after direct PRL), and (**d**) S-C/SiO_x_-Si-C full cell (after electrochemical PRL).

In order to further examine the cycling performance of the Li-S full cells with pre-lithiated SiO_x_-Si-C composite anodes, a Li-S half-cell with lithium metal anode was evaluated. The S-C cathode was prepared with 60% sulfur. Figure S2a–c, respectively, depict the XRD patterns of the elemental sulfur, MWCNTs, and sulfur-impregnated MWCNTs. The disappearance of peaks of elemental sulfur in Figure S2c confirmed the confinement of elemental sulfur within the MWCNTs, and the amount of elemental sulfur was calculated to be 60% according to the TG analysis (Figure S2d). The areal loading of sulfur was calculated to be 2.5 mg cm^−2^. The amount of non-aqueous liquid electrolyte was 6 mL/cm^2^ of sulfur. The galvanostatic charge–discharge profiles were studied for up to 20 cycles and are shown in Figure 8a. The upper plateau region that appeared between 2.3 and 2.5 V illustrates the conversion of active materials from cyclic S_8_ to soluble polysulfides (Li_2_S_n_, 4 < n ≤ 8), while the second lower plateau represents the further reduction of Li_2_S_8_ to Li_2_S_2_ or Li_2_S. Subsequently, during the charging process, Li_2_S was delithated to form Li_2_S_4_ and finally S_8_. The plateaus were very flat and had relatively low polarization at the present current rate (0.1C), which indicates a kinetically efficient reaction.

The Li-S cell delivered a discharge capacity of 795 and 765 mAh g^−1^ on its 1st and 20th cycles, respectively, with a capacity fade per cycle equal to 1.5 mAh g^−1^. The cycling performance of a Li-SiO_x_-Si-C composite electrode (pre-lithiated for 30 min by direct contact) is shown in Figure 8b. The cell offered a discharge capacity of 1359 and 752 mAh g^−1^ on its 1st and 20th cycles, respectively, with a fade in capacity per cycle of 30 mAh g^−1^.

**Figure 9:**
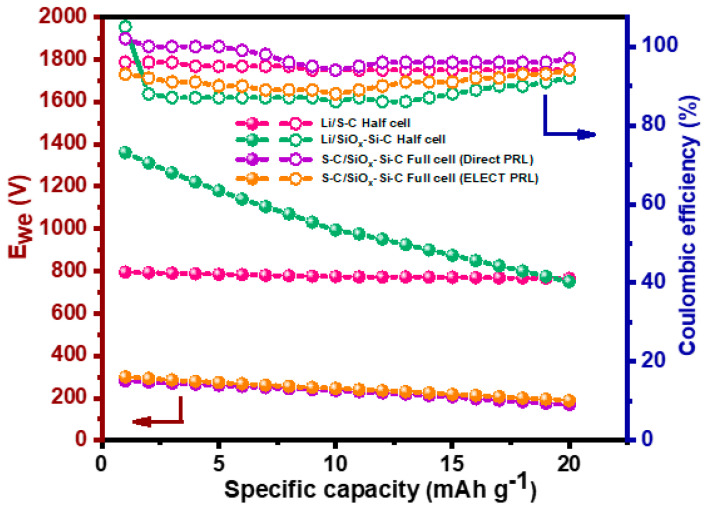
Discharge capacity and Coulombic efficiency vs. cycle number.

Figure 8c,d illustrate the cycling performance of S-C and S-C/SiO_x_-Si-C composite electrode full cells pre-lithiated using direct contact and electrochemical methods, respectively. The open circuit voltages of the S-C/SiO_x_-Si-C composite with pre-lithiated anodes were 2.19 and 2.1 V, respectively. Figure 9 shows the discharge capacity vs. cycle number for S-C/SiO_x_-Si-C composite anode full cells. It is clear from Figure 8c,d that the S-C/SiO_x_-Si-C full cell with an electrochemically pre-lithiated SiO_x_-Si-C composite anode offered a higher discharge capacity than the anode pre-lithiated using the direct contact method. The S-C/SiO_x_-Si-C full cell with an electrode pre-lithiated using direct contact offered a discharge capacity of 281 and 169 mAh g^−1^ on its 1st and 20th cycles, respectively, with a capacity fade per cycle of 5.6 mAh g^−1^. On the other hand, the S-C/SiO_x_-Si-C full-cell pre-lithiated electrode produced by an electrochemical method delivered a discharge capacity of 300 and 189 mAh g^−1^ on its 1st and 20th cycles, respectively, with a capacity fade per cycle of 5.6 mAh g^−1^. Although few variations in the discharge capacity were seen in both methods, the Columbic efficiency for the S-C/SiO_x_-Si-C full cell with an electrode pre-lithiated using a direct contact method was higher than that with an electrochemically pre-lithiated electrode, as shown in Figure 8. The lower discharge capacity of the S-C/SiO_x_-Si-C full cell was attributed to an inappropriate balance between the anode and composite sulfur cathode without any catalyst and with non-optimized amounts of liquid electrolytes.

## 4. Conclusions

In summary, SiO_x_-Si-C composite electrodes were prepared with different amounts of SiO_x_, Si, and C. The composite electrode with a composition of SiO_x_:Si:C of 70:12.5:12.5 was optimal and delivered a maximum discharge capacity of 698 mAh g^−1^, even after 100 cycles. The SEM analyses additionally demonstrated a uniform and unbroken surface morphology at a 75% graphitic carbon content. In contrast, when the silicon content was high, the surface morphology of the electrode was fractured. On the other hand, the XPS spectra showed no additional peaks, except one at 689.09 eV, which represents the formation of –CF_2_. Further, pre-lithiation of SiO_x_-Si-C electrodes was performed using both electrochemical and direct contact methods. The S-C/SiO_x_-Si-C full-cell studies with SiO_x_-Si-C composite anodes pre-lithiated using an electrochemical method offered a higher discharge capacity than the anodes pre-lithiated using the direct contact method. The areal loading of sulfur in the cathode was 2.5 mg cm^−2^. The poor discharge capacity of S-C/SiO_x_-Si-C full cells was attributed to the imbalance in active materials in the cathode and anode, which is being studied in our laboratory and the results will be communicated in due course.

## Figures and Tables

**Table 1:** Formulations containing different amounts of SiO_x_, Si, and graphitic carbon. Binder was used to bring the total % up to 100%.

Formulation	SiO_x_ (wt.%)	Si (wt.%)	Graphitic Carbon (wt.%)
a	85	0.0	10.0
b	75	10	10.0
c	70	12.5	12.5
d	65	20	10.0
e	10	75	10.0
f	0.0	85	10.0
g	10	10	75

## Data Availability

The original contributions presented in this study are included in the article/Supplementary Material. Further inquiries can be directed to the corresponding authors.

## Supplementary Materials

The following supporting information can be downloaded at: https://www.mdpi.com/article/10.3390/ma18051053/s1. Figure S1. Charge-discharge profiles of Li/SiO_x_-Si-C cells with different compositions (a–g), the details of which are shown in Table S1. Figure S2. XRD spectra of (a) elemental sulfur, (b) sulfur and MWCNT composite heated at 155 °C/12 h, (c) sulfur and MWCNT composite heated at 300 °C/12 h. (d) TG analysis of sulfur and MWCNT composite (S:MWCNT) melt-diffused at 300 °C for 12 h under N_2_ atmosphere. Figure S3. Cyclic voltammograms of Li/SiO_x_-Si-C cells with different compositions at 0.05 mV s^−1^. Figure S4. Discharge capacity as a function of cycle number at 0.1C rate with different compositions. Figure S5. XPS spectra of sample b (75% SiO_x_ + 10% Si + 10% C) after 100 cycles. Table S1. Electrochemical performance of SiO*_x_*-Si-C with different compositions. Table S2. EIS parameters obtained for SiO*_x_*-Si-C samples with different compositions.

## References

[1] Tarascon J.-M., Armand M. (2001). Issues and challenges facing rechargeable lithium batteries. Nature.

[2] Yoo H.D., Markevich E., Salitra G., Sharon D., Aurbach D. (2014). On the challenge of developing advanced technologies for electrochemical energy storage and conversion. Mater. Today.

[3] Cleaver T., Kovacik P., Marinescu M., Zhang T., Offer G. (2018). Perspective-Commercializing lithium sulfur batteries: Are we doing the right research?. J. Electrochem. Soc..

[4] Ji X., Nazar L.F. (2010). Advances in Li-S batteries. J. Mater. Chem..

[5] Yang Y., Zheng G., Cui Y. (2013). Nanostructured sulfur cathodes. Chem. Soc. Rev..

[6] Suriyakumar S., Stephan A.M. (2020). Mitigation of polysulfide shuttling by interlayer/permselective separators in lithium-sulfur batteries. ACS Appl. Energy Mater..

[7] Rosenman A., Markevich E., Salitra G., Aurbach D., Garsuch A., Chesneau F.F. (2015). Review on Li-sulfur battery systems: An integral perspective. Adv. Energy Mater..

[8] Zhao X., Kim D.-S., Ahn H.-J., Kim K.-W., Cho K.-K., Ahn J.-H. (2014). Infiltrating sulfur into a highly porous carbon sphere as cathode material for lithium–sulfur batteries. Mater. Res. Bull..

[9] Wu H., Chan G., Choi J.W., Ryu I., Yao Y., McDowell M.T., Lee S.W., Jackson A., Yang Y., Hu L. (2012). Stable cycling of double-walled silicon nanotube battery anodes through solid-electrolyte interphase control. Nat. Nanotechnol..

[10] Kim H., Han B., Choo J., Cho J. (2008). Three-dimensional porous silicon particles for use in high-performance lithium secondary batteries. Angew. Chem. Int. Ed..

[11] Cao R., Xu W., Lv D., Xiao J., Zhang J.-G. (2015). Anodes for rechargeable lithium-sulfur batteries. Adv. Energy Mater..

[12] Zuo X., Zhu J., Muller-Buschbaum P., Cheng Y.-J. (2017). Silicon based lithium-ion battery anodes: A chronicle perspective review. Nano Energy.

[13] Jin Y., Li S., Kushima A., Zheng X., Sun Y., Xie J., Sun J., Xue W., Zhou G., Wu J. (2017). Self-healing SEI enables full-cell cycling of a silicon-majority anode with a coulombic efficiency exceeding 99.9%. Energy Environ. Sci..

[14] Xu Q., Li J.-Y., Sun J.-K., Yin Y.-X., Wan L.-J., Guo Y.-G. (2017). Watermelon-inspired Si/C microspheres with hierarchical buffer structures for densely compacted lithium-ion battery anodes. Adv. Energy Mater..

[15] Zhou X., Yin Y.X., Wan L.J., Guo Y.G. (2012). Facile synthesis of silicon nanoparticles inserted into graphene sheets as improved anode materials for lithium-ion batteries. Chem. Commun..

[16] Bao Z., Ernst E.M., Yoo S., Sandhage K. (2009). Syntheses of porous self-supporting metal-nanoparticle assemblies with 3D morphologies inherited from Biosilica templates (diatom frustules). Adv. Mater..

[17] Dalavi S., Guduru P., Lucht B.L. (2012). Performance enhancing electrolyte additives for lithium-ion batteries with silicon anodes. J. Electrochem. Soc..

[18] Higgins T.M., Park S.-H., King P.J., Zhang C.J., McEvoy N., Berner N.C., Daly D., Shmeliov A., Khan U., Duesberg G. (2016). A commercial conducting polymer as both binder and conductive additive for silicon nanoparticle-based lithium-ion battery negative electrodes. ACS Nano.

[19] Liu Z., Yu Q., Zhao Y., He R., Xu M., Feng S., Li S., Zhou L., Mai L. (2019). Silicon oxides: A promising family of anode materials for lithium-ion batteries. Chem. Soc. Rev..

[20] Choi I., Lee M.J., Oh S.M., Kim J.J. (2012). Fading mechanisms of carbon-coated and disproportionated Si/SiO*_x_* negative electrode (Si/SiO*_x_*/C) in Li-ion secondary batteries: Dynamics and component analysis by TEM. Electrochim. Acta.

[21] Yang C., Zhang Y., Zhou J., Lin C., Lv F., Wang K., Feng J., Xu Z., Li J., Guo S. (2018). Hollow Si/SiO_x_ nanosphere/nitrogen-doped carbon superstructure with a double shell and void for high-rate and long-life lithium-ion storage. J. Mater. Chem. A.

[22] Chen T., Wu J., Zhang Q., Su X. (2017). Recent advancement of SiO_x_ based anodes for lithium-ion batteries. J. Power Sources.

[23] Choi N.-S., Yew K.H., Lee K.Y., Sung M., Kim H., Kim S.-S. (2006). Effect of fluoroethylene carbonate additive on interfacial properties of silicon thin-film electrode. J. Power Sources.

[24] Tang J., Hou L., Hu T., Fan S., Zhou X., Yang J. (2021). Influence of oxygen content on the electrochemical behavior of SiO_x_@C anodes for Li-ion battery. Compos. Commun..

[25] Zhang L., Deng J., Liu L., Si W., Oswald S., Xi L., Kundu M., Ma G., Gemming T., Baunack S. (2014). Hierarchically designed SiO_x_/SiO_y_ bilayer nanomembranes as stable anodes for lithium ion Batteries. Adv. Mater..

[26] Sun M., Ma J., Xu M., Yang H., Zhang J., Wang C. (2022). A low-cost SiO*_x_*/C@Graphite composite derived from oat husk as an advanced anode for high-performance lithium-ion Batteries. ACS Omega.

[27] Hagen M., González E.Q., Dörfler S., Fahrer G., Tübke J., Hoffmann M.J., Althues H., Speck R., Krampfert M., Kaskel S. (2014). Studies on preventing Li dendrite formation in Li-S batteries by using pre-lithiated Si microwire anodes. J. Power Sources.

[28] Rahman I.A., Padavettan V. (2012). Synthesis of Silica nanoparticles by sol-gel: Size-dependent properties, surface modification, and applications in silica-polymer nanocompositesa review. J. Nanomater..

[29] Liu Y., Pelster T., Lee T.-T., Wang Y., Luo G. (2022). Study on the three-stage growth of silica nanoparticles prepared by the drop-by-drop precipitation method. Powder Technol..

[30] Qi D., Lin C., Zhao H., Liu H., Lü T. (2017). Size regulation and prediction of the SiO_2_ nanoparticles prepared via Stöber process. J. Dispersion Sci. Technol..

[31] Sathya S., Soosaimanickam C., Bella F., Yoo D.J., Stephan A.M. (2024). Cycling performance of SiO_x_-Si-C composite anode with different blend ratios of PAA-CMC as binder for lithium sulfur batteries. J. Polym. Res..

[32] Sathya S., Angulakshmi N., Ahn J.-H., Kathiresan M., Stephan A.M. (2022). Influence of additives on the electrochemical and interfacial properties of SiO_x_-based anode materials for lithium–sulfur batteries. Langmuir.

[33] Guo C., Wang D., Liu T., Zhu J., Lang X. (2014). A three dimensional SiOx/C@RGO nanocomposite as a high energy anode material for lithium-ion batteries. J. Mater. Chem. A.

[34] Pollak E., Salitra G., Baranchugov V., Aurbach D. (2007). In situ conductivity, impedance spectroscopy, and ex situ Raman spectra of amorphous silicon during the insertion/extraction of lithium. J. Phys. Chem. C.

[35] Netz A., Huggins R.A., Weppner W. (2003). The formation and properties of amorphous silicon as negative electrode reactant in lithium systems. J. Power Sources.

[36] Minakshi M., Mitchell D.R.G., Munnangi A.R., Barlow A.J., Fichtner M. (2018). New insights into the electrochemistry of magnesium molybdate hierarchical architectures for high performance sodium devices. Nanoscale.

[37] Sharma P., Minakshi Sundaram M., Watcharatharapong T., Laird D., Euchner H., Ahuja R. (2020). Zn metal atom doping on the surface plane of one-dimesional NiMoO_4_ nanorods with improved redox chemistry. ACS Appl. Mater. Interfaces.

[38] Schroder K.W., Celio H., Webb L.J., Stevenson K.J. (2012). Examining solid electrolyte interphase formation on crystalline silicon electrodes: Influence of electrochemical preparation and ambient exposure conditions. J. Phys. Chem. C.

[39] Etacheri V., Haik O., Goffer Y., Roberts G.A., Stefan I.C., Fasching R., Aurbach D. (2012). Effect of fluoroethylene carbonate (FEC) on the performance and surface chemistry of Si-nanowire Li-ion battery anodes. Langmuir.

[40] Yamin H., Peled E. (1983). Electrochemistry of a nonaqueous lithium/sulfur cell. J. Power Sources.

[41] Ruffo R., Hong S.S., Chan C.K., Huggins R.A., Cui Y. (2009). Impedance analysis of silicon nanowire lithium ion battery anodes. J. Phys. Chem. C.

[42] Chakrapani V., Rusli F., Filler M.A., Kohl P.A. (2011). Quaternary ammonium ionic liquid electrolyte for a silicon nanowire-based lithium ion battery. J. Phys. Chem. C.

[43] Jiang T., Zhang S., Qiu X., Zhu W., Chen L. (2007). Preparation and characterization of silicon-based three-dimensional cellular anode for lithium-ion battery. Electrochem. Commun..

[44] Eriksson T., Andersson A.M., Gejke C., Gustafsson T., Thomas J.O. (2002). Influence of temperature of the interface chemistry of Li_x_Mn_2_O_4_ electrodes. Langmuir.

[45] Jia H., Zou L., Gao P., Cao X., Zhao W., He Y., Engelhard M.H., Burton S.D., Wang H., Ren X. (2019). High-performance silicon anodes enabled by nonflammable localized high-concentration electrolytes. Adv. Energy Mater..

[46] Diao Y., Xie K., Xiong S., Hong X. (2012). Insights into Li-S battery cathode capacity fading mechanisms: Irreversible oxidation of active mass during cycling. J. Electrochem. Soc..

[47] Jiang L., Park-Lee K.J., Clinton R.M., Tang Z., Breedveld V., Hess D.W. (2017). Mechanical durability of liquid repellent coatings. Surf. Coat. Technol..

[48] Gnanaraj J.S., Levi M.D., Levi E., Salitra G., Aurbach D., Fischer J.E., Claye A. (2001). Comparison between the electrochemical behavior of disordered carbons and graphite electrodes in connection with their structure. J. Electrochem. Soc..

[49] Chen X., Wang X., Fang D. (2020). A review on C1s XPS-spectra for some kinds of carbon materials. Fuller. Nanotub. Carbon Nanostruct..

[50] Dornbusch M. (2018). Chapter IV. Corrosion Analysis.

[51] Marinaro M., Weinberger M., Mehrens M. (2016). Toward pre-lithiated high areal capacity silicon anodes for lithium-ion batteries. Electrochim. Acta.

[52] Zhang J., Shi Z., Wang C. (2014). Effect of Pre-lithiation degrees of mesocarbon microbeads anode on the electrochemical performance of lithium-ion capacitors. Electrochim. Acta.

[53] Kim H.S., Jeoung T.-G., Kim Y.-T. (2016). Electrochemical properties of lithium sulfur battery with silicon anodes lithiated by direct contact method. J. Electrochem. Sci. Technol..

[54] Holtstiege F., Bärmann P., Nölle R., Winter M., Placke T. (2018). Pre-lithiation strategies for rechargeable energy storage technologies: Concepts, promises and challenges. Batteries.

[55] Domi Y., Usui H., Iwanari D., Sakaguchi H. (2017). Effect of Mechanical pre-lithiation on electrochemical performance of silicon negative electrode for lithium-ion batteries. J. Electrochem. Soc..

[56] Chang S., Moon J., Cho K., Cho M. (2015). Multiscale analysis of prelithiated silicon nanowire for Li-ion battery. Comput. Mater. Sci..

[57] Shellikeri A., Watson V.G., Adams D.L., Kalu E.E., Read J.A., Jow T.R., Zheng J.P. (2017). Pre-lithiation of carbon anodes using different lithium–sources. ECS Trans..

[58] Zhou J., Lu Y., Yang L., Zhu W., Liu W., Yang Y., Liu K. (2022). Sustainable silicon anodes facilitated via a double-layer interface engineering: Inner SiOx combined with outer nitrogen and boron co-doped carbon. Carbon Energy.

[59] Jiang R., Yuan H., Wei X., Wang H., Shin H.J., Lan J., Yu Y., Yang Y. (2021). Constructing robust and freestanding MXene/Si@C core–shell nanofibers via coaxial electrospinning for high performance Li-ion batteries. Mater. Chem. Front..

