# Supplementary material for: Interplay Between Composition and Cycling Performance of Pre-Lithiated SiOx-Si-C Composite Anodes for Lithium–Sulfur Full Cells
Source: Materials (Basel). 2025 Feb 27;18(5):1053. doi: 10.3390/ma18051053 (PMC11901105; doi:10.3390/ma18051053)
Supplement: Supplementary file 1 [file materials-18-01053-s001.zip › materials-3442913-supplementary.pdf]

Supporting Information

Interplay Between Composition and Cycling Performance of Pre-Lithiated SiOx-Si-C Composite Anodes for Lithium–Sulfur Full Cells

Swamickan Sathya [1], Ramasamy Santhosh Kumar [2], Sara Garcia-Ballesteros [3], Federico Bella [3,*], Dong Jin Yoo [2,*] and Arul Manuel Stephan [1,*]

[1] Electrochemical Power Sources Division, CSIR-Central Electrochemical Research Institute, Karaikudi 630003, India

[2] Department of Life Science, Department of Energy Storage/Conversion Engineering of Graduate School (BK21 FOUR), Hydrogen and Fuel Cell Research Center, Jeonbuk National University, 567 Baekje-daero, Jeonju 54896, Jeollabuk-do, Republic of Korea

[3] Department of Applied Science and Technology, Politecnico di Torino, Corso Duca degli Abruzzi 24, 10129-Torino, Italy

* Correspondence: federico.bella@polito.it (F.B.); djyoo@jbnu.ac.kr (D.J.Y.); arulmanuel@gmail.com (A.M.S.)

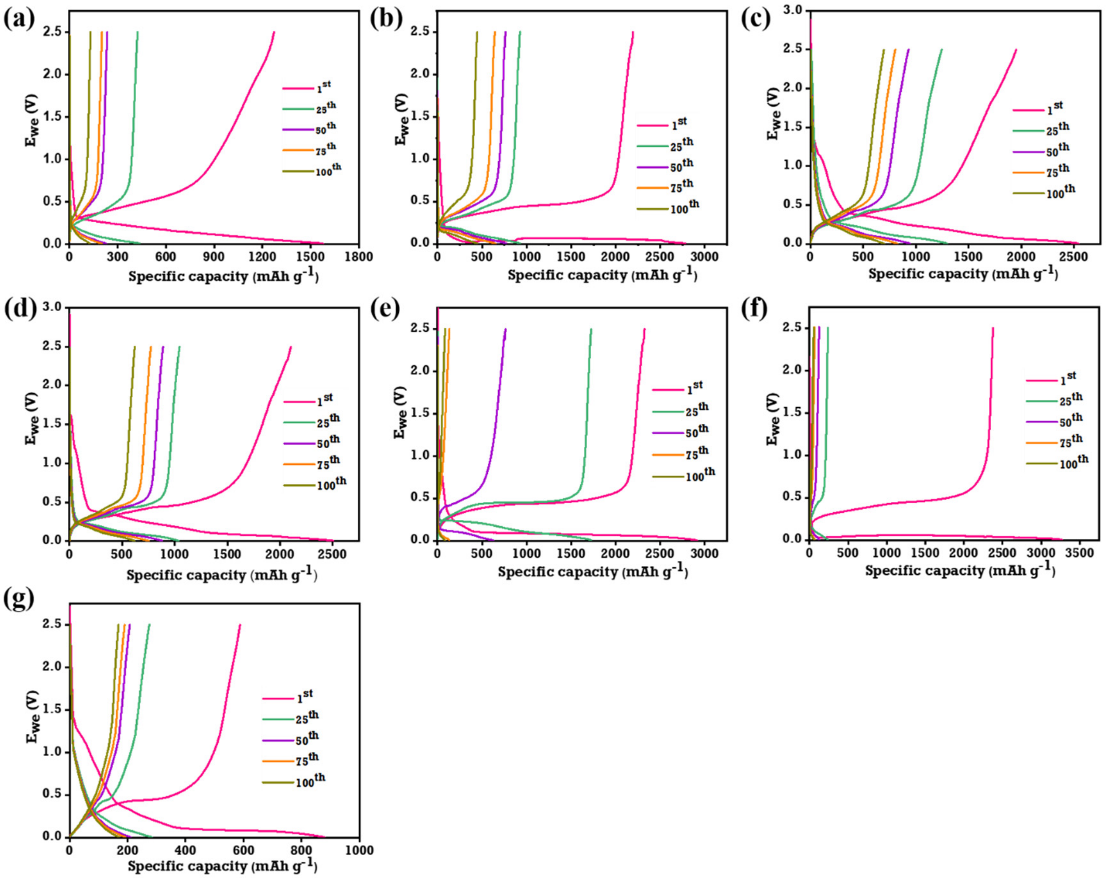

Figure S1. Charge-discharge profiles of Li/SiO_x-Si-C cells with different compositions (a-g), the details of which are shown in Table 1.

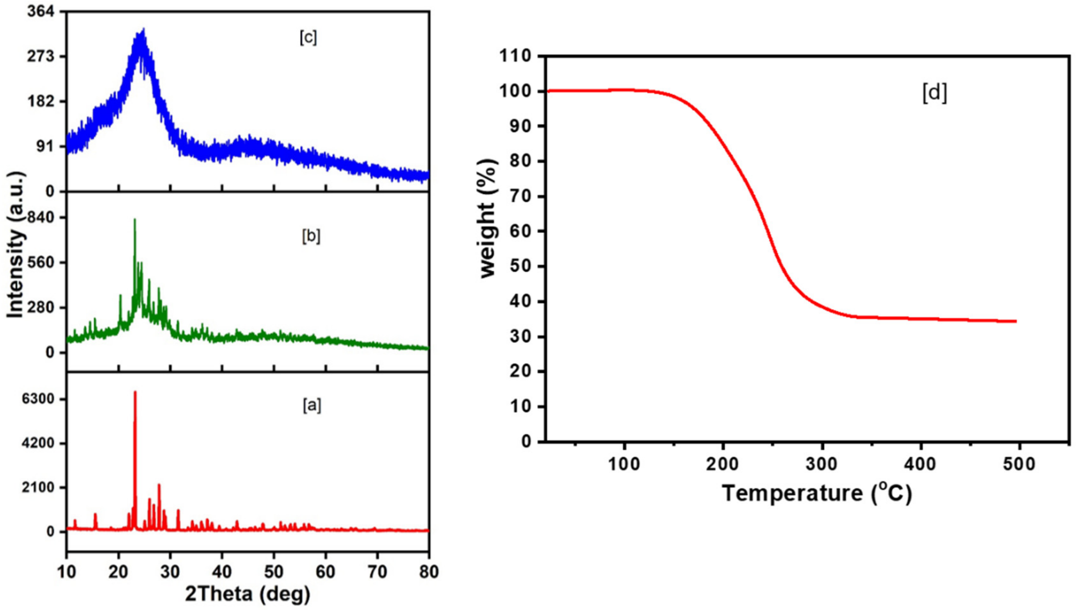

Figure S2. XRD spectra of (a) elemental sulfur, (b) sulfur and MWCNT composite heated at 155 °C/12 h, (c) sulfur and MWCNT composite heated at 300 °C/12 h. (d) TG analysis of sulfur and MWCNT composite (S:MWCNT) melt-diffused at 300 °C for 12 h under N_2 atmosphere.

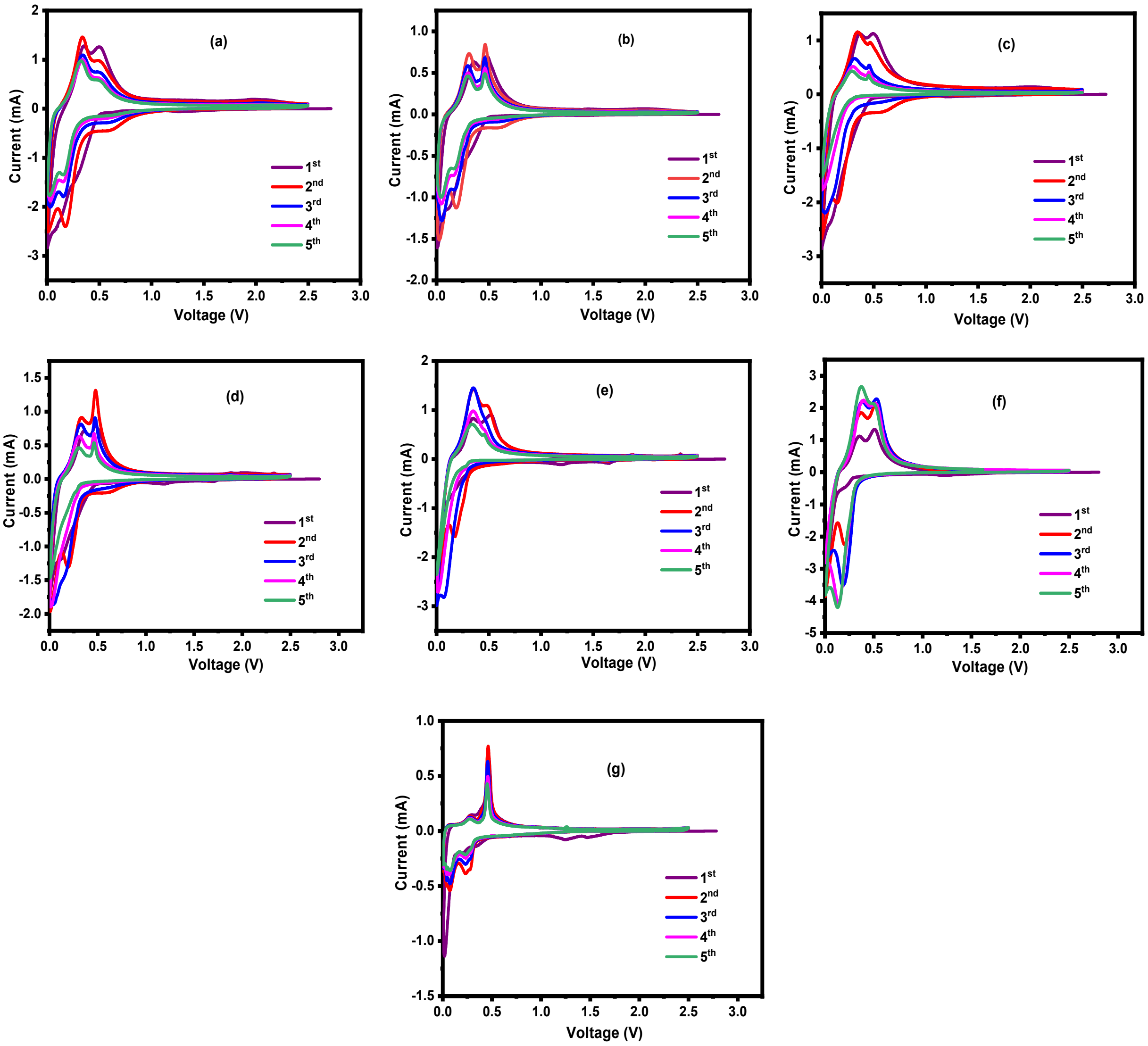

Figure S3. Cyclic voltammograms of Li/SiO_x-Si-C cells with different compositions at 0.05 mV s^{-1}.

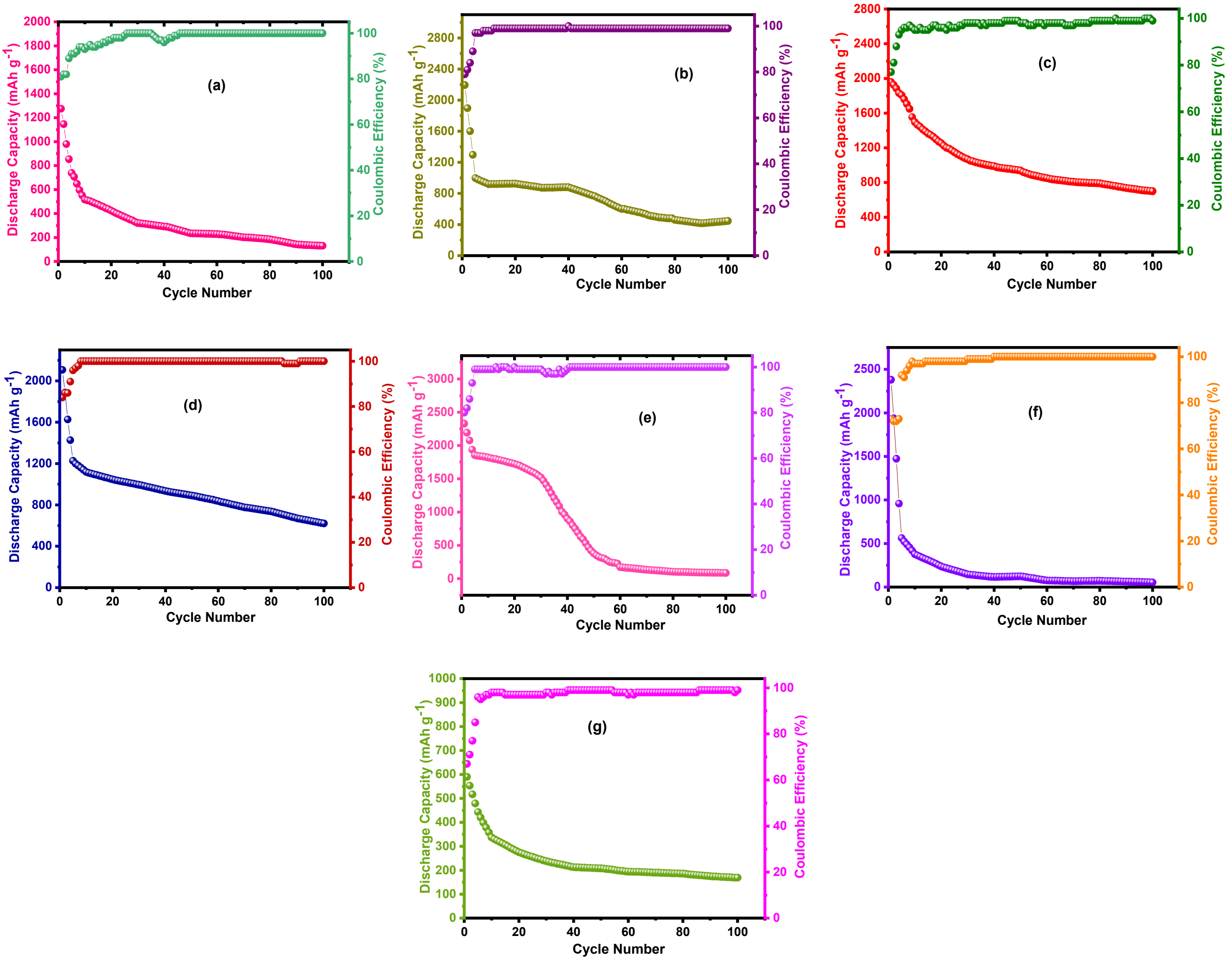

Figure S4. Discharge capacity as a function of cycle number at 0.1C rate with different compositions.

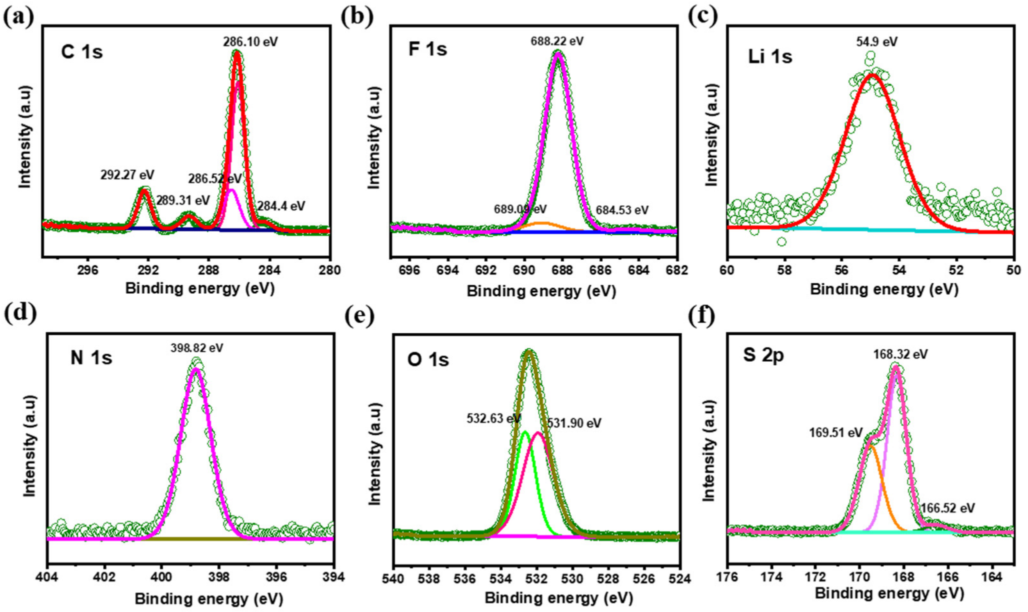

Figure S5. XPS spectra of sample b (75% SiO_x + 10% Si + 10% C) after 100 cycles.

Table S1. Electrochemical performance of SiO_x-Si-C with different compositions.

Sample	Specific capacity (1st cycle)			Specific capacity (50th cycle)			Specific capacity (100th cycle)		
	C (mAh g^{-1})	D (mAh g^{-1})	CE (%)	C (mAh g^{-1})	D (mAh g^{-1})	CE (%)	C (mAh g^{-1})	D (mAh g^{-1})	CE (%)
a	1576	1272	81	232	232	100	129	129	100
b	2784	2195	79	771	762	99	446	446	100
c	2542	1952	77	946	931	98	701	698	99
d	2508	2106	84	890	890	100	621	621	100
e	2913	2323	80	766	766	100	85	85	100
f	3270	2374	73	124	123	99	54	54	100
g	880	588	67	210	207	99	171	169	99

Table S2. EIS parameters obtained for SiO_x-Si-C samples with different compositions.

Composition	R_s			R_{SEI}			R_{ct}		
	Before	50th	100th	Before	50th	100th	Before	50th	100th
a	4	6	5	-	4	5	20	5	8
b	6	4	16	-	5	4	74	8	9
c	5	13	25	-	10	11	38	11	12
d	5	4	15	-	9	27	30	11	57
e	4	5	23	-	16	22	27	24	60
f	4	15	24	-	22	30	56	40	83
g	4	4	5	-	6	5	70	9	11
